# The use of a novel cleaning closed suction system reduces the volume of secretions within the endotracheal tube as assessed by micro-computed tomography: a randomized clinical trial

**DOI:** 10.1186/s13613-015-0101-9

**Published:** 2015-12-30

**Authors:** Andrea Coppadoro, Giacomo Bellani, Alfio Bronco, Alberto Lucchini, Simone Bramati, Vanessa Zambelli, Roberto Marcolin, Antonio Pesenti

**Affiliations:** Department of Health Science, University of Milan-Bicocca, Via Cadore 48, Monza (MB), 20900 Italy; Department of Anesthesia and Intensive Care, San Gerardo Hospital, Monza, Italy; Laboratory of Microbiology, San Gerardo Hospital, Monza, Italy; A.Manzoni Hospital, Lecco, Italy

**Keywords:** Endotracheal tube, Cleaning closed suction system, Biofilm, Resistance to airflow, Secretion volume, Cross-sectional area

## Abstract

**Background:**

Early after intubation, a layer of biofilm covers the inner lumen of the endotracheal tube (ETT). Cleaning the ETT might prevent airways colonization by pathogens, reduce resistance to airflow, and decrease sudden ETT obstruction. We investigated the effectiveness of a cleaning closed suction system in maintaining the endotracheal tube free from secretions.

**Methods:**

We conducted a single center, randomized controlled trial, in the general intensive care unit of a tertiary-level university hospital. We enrolled 40 adult critically ill patients expected to remain intubated for more than 48 h, within 24 h from intubation. Patients were randomized to receive three ETT cleaning maneuvers/day using a novel device (Airway Medix Closed Suction System™, cleaning group) or to standard care (no ETT cleaning, standard closed suction, control group). After extubation, the amount of secretions in the ETTs was measured by micro-computed tomography.

**Results:**

The volume of secretions in the ETTs from the cleaning group was lower than controls (0.081 [0.021–0.306] vs. 0.568 [0.162–0.756] mL, *p* = 0.001), corresponding to a cross-sectional area reduction six times lower (1[0–3] vs. 6 [2–10] %, *p* = 0.001). In a subset of 16 patients, the resistance to airflow tended to be lower after 1 day of treatment (*p* = 0.063) and was lower after 2 days (0.024), while no difference was present at enrollment (*p* = 0.922). ETT colonization did not differ between the two groups.

**Conclusions:**

The use of a novel cleaning closed suction system proved to be effective in reducing secretions present in the ETT after extubation, possibly reducing resistance to airflow during intubation.

Trial registration: clinicaltrials.gov NCT01912105

**Electronic supplementary material:**

The online version of this article (doi:10.1186/s13613-015-0101-9) contains supplementary material, which is available to authorized users.

## Background

Endotracheal intubation is a necessary choice to ventilate many critically ill patients; however, the use of an endotracheal tube (ETT) is associated with several complications [1]. The presence of secretions within the ETT has been advocated in the pathogenesis of ventilator-associated pneumonia (VAP) since many years [2]. Soon after intubation, in the ETT is detectable a layer of biofilm composed by mucosal secretions, erythrocytes, neutrophils, and possibly microbes [3]. The ETT biofilm thickness increases with time and airflow can transport microbial aggregates towards the distal airways, leading to pneumonia [4]. The link between ETT biofilm and VAP has been well established: the prevention of biofilm formation or its removal through modified ETTs has been associated with reduced incidence of VAP in the clinical scenario [5]. Standard care is not adequate to completely remove secretions from the ETT and several additional measures aimed to reduce ETT biofilm have been proposed to prevent VAP [6].

The presence of secretions within the ETT is also potentially associated with obstruction and increased resistance to airflow. Specifically designed devices (i.e., the Mucus Shaver) are able to mechanically remove secretions from the inner ETT surface, maintaining ETT patency and reducing ETT bacterial colonization [7]. In difficult to wean patients, restoring airway resistance to nominal values through ETT secretion removal might represent a clinically relevant advantage.

In this randomized trial, we evaluated the efficacy of a cleaning closed suction system (CSS), a device designed to combine a closed suction catheter and an ETT water-based cleaning system. The device is designed to be used several times for all the duration of intubation, with the aim of keeping the ETT free from secretions and therefore the potential advantage of lower ETT microbial colonization and lower resistance to airflow. We hypothesized that patients treated with the cleaning CSS would have, at the moment of extubation, a lower amount of secretions within the ETT as compared to controls treated with a standard CSS. We measured the volume of secretions present in the ETT after extubation using Micro-computed tomography (MicroCT), a reliable technique able to measure the amount of secretions of a large part of the ETT [8].

## Methods

### Ethics, consent, and permissions

We conducted a controlled randomized single-blinded clinical trial (clinicaltrials.gov: NCT01912105) in the general intensive care unit (ICU) of a tertiary-care university hospital (San Gerardo Hospital, Monza, Italy). The study was approved by the local Institutional Review Board (San Gerardo Hospital Ethical Committee) and was carried out in compliance with the Helsinki Declaration. Request of written informed consent was delayed until patient recovery and family members were informed, as per local regulation.

### Patients

**Fig. 1:**
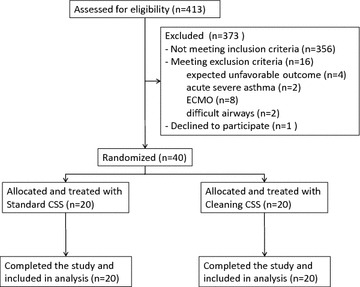
The CONSORT diagram for the study procedures. A total of 40 patients were randomized to receive ETT cleaning (treatment group) or standard care (controls). *ECMO* extracorporeal membrane oxygenation; *CSS* closed suction system

From April 2013 to June 2014, forty patients were randomized and successfully completed the study (see Fig. 1). Inclusion criteria were as follows: age >18 years old, intubation within 24 h, expected intubation longer than 48 h, and FiO_2_ <80 % at the moment of patients screening. Exclusion criteria were as follows: current or past participation in another intervention trial conflicting with the present study, expected survival less than 24 h or high probability of unfavorable outcome (e.g., return of spontaneous circulation after unwitnessed cardiac arrest, no-flow >10 min or age >65), acute severe asthma, extracorporeal membrane oxygenation treatment, presence of double lumen ETT, known difficult airway management in case of ETT displacement (such as upper airway edema, or cervical spine trauma), and contraindication posed by staff physicians. Patients enrolled in the study who were extubated and required reintubation were not enrolled in the study a second time.

### Study protocol

Upon enrollment, patients were randomized to receive standard care (control group) with the CSS in use in our ICU (KimVent closed suction system, Kimberly-Clark Corp., Neenah, WI USA,) or the use of a novel cleaning CSS (Airway Medix closed suctioning system, Biovo Technologies, Tel Aviv, Israel, treatment group). A computer-based random sequence was used to obtain a 1:1 randomization ratio in blocks of 10 patients. The cleaning CSS used in the treatment group is a suction catheter with aspiration holes on the side and a balloon on its tip, which normally remains deflated and can be used to perform a standard suction maneuver. When the cleaning option is activated, the same device can also be used to clean the ETT: the catheter is inserted within the ETT similarly to a standard suction maneuver, and then the activation of the suction channel occurs together with the inflation of the cleaning balloon and the activation of water jets. When the catheter is gently retracted, the inflated balloon and the water jets gather secretions from the ETT wall, which are removed by the aspirating system.

A standard CSS was used in the control group, while in the treatment group tracheal suction was performed with the study CSS with the cleaning option disabled. In the treatment group, three additional cleaning maneuvers were performed every day (once every shift). Tracheal suction maneuvers were performed by critical care nurses when needed, based on the standard protocol in use in our ICU with the aim of a secretion detector [9], both in the treatment and in the control groups. Further details about airways management can be found in the supplemental digital content (Additional file 1).

### Data collection

Patient’s history and demographics were recorded, together with several clinical parameters, investigating the presence of Acute Respiratory Distress Syndrome diagnosis [10] and assessing the Simplified Acute Physiology Score II [SAPS2] [11].

We collected daily clinical data regarding ventilator parameters and signs of infection, to calculate a modified clinical pulmonary infection score (mCPIS) [12]. Nurses recorded the total number of tracheal suction maneuvers performed, along with major adverse events related to the use of the new cleaning device. These were defined as follows: desaturation (drop in SpO2 >5 %) persisting for more than 5 min after suction and requiring a change in ventilator parameters; modification of blood pressure (systolic >200 mmHg or diastolic <80 mmHg if not already present) requiring new drug therapy; persistent reduction of ETCO2 (drop >5 mmHg); persistence of tachycardia or occurrence of other arrhythmia following the cleaning maneuver; ETT displacement requiring reintubation. When planned in advance, we collected surveillance tracheal aspirate samples in the 24 h prior to extubation. At extubation, we recorded ICU length of stay, ventilator free days in the first 28 days, and patient outcome. We also assessed the incidence of VAP and ventilator-associated events (VAE). Further details about data collection are provided in the Additional file 1.

### Sample processing

After extubation, an investigator blind to the randomization group performed a MicroCT scan of the collected ETTs (SkyScan 1176, Bruker, Belgium) for a length of 20 cm from the ETT tip. After image reconstruction, an automated software (CT Analyzer, Bruker, Belgium) analyzed the CT images based on densitometric criteria, to obtain the measurement of the total volume of secretions and the ETT lumen cross-sectional area reduction due to the presence of secretions. Image analyses were conducted using the MicroCT scan embedded software (CT Analyzer, Bruker, Belgium). To describe the local distribution of the secretions within the ETT, we calculated the average amount of secretions and cross-sectional area reduction every 0.5 mm. To analyze possible differences of the regional distribution of the secretions between the two groups, the same variables were also calculated dividing the ETT length in three equal parts: ventilator side, central, and tip side. After MicroCT scan, ETT microbial colonization was assessed (see Additional file 1 for details).

### Statistical methods

We based our sample size estimation on previous explorative laboratory analyses (unpublished data) investigating the volume of secretions present within ETTs after extubation by CT scan. We hypothesized an average secretions volume of 2.1 ± 2.1 ml in the control group, and 0.2 ml in the treatment group. Therefore, a sample size of 20 patients per arm (total 40 enrolled patients) would provide 80 % power to detect a difference considering a *p* value level of 0.05 as significant.

Normality of variables’ distribution was assessed by Shapiro–Wilk’s test; normally and non-normally distributed data are presented as mean ± SD and median [inter-quartile range], respectively. Differences between the two groups were tested by Student’s t-test for normally distributed variables, and by Mann–Whitney test for non-normally distributed variables. Categorical variables’ differences were analyzed by Fisher’s exact test in case of dichotomous variables, otherwise by Chi-Square test. To evaluate differences of secretion distribution within the ETT, a Repeated Measures ANOVA model was performed, considering the randomization group as between-subject factor, and the regional distribution (three ETT parts) as within-subject factor; Tukey’s correction for post hoc tests was used. Statistical analyses were performed using SPSS software version 18.0 (Chicago, IL) and SigmaPlot 11 (Systat software Inc., Germany). A *p* value <0.05 was considered statistically significant.

## Results

Forty patients were studied; 26 were successfully extubated, 12 died and ETTs were removed post-mortem, one had an unplanned extubation, one had a failed extubation and required reintubation, and none underwent tracheostomy. In total, we collected data on 252 ventilator days; nearly a thousand of tracheal suction maneuvers and more than 300 cleaning maneuvers were performed during the study. The nurses in charge of the patients did not report any relevant adverse event related to the use of the cleaning CSS, neither reported any major issue that prevented the correct use of the device. Sudden ETT occlusion requiring emergent bronchoscopy occurred in one patient in the treatment group and one patient in the control group; in both cases, after bronchoscopy, the ETTs were removed and the patients were re-intubated.

The control and the treatment groups were similar at enrollment, although patients in the treatment group tended to be more severe as showed by SAPS2 on admission (Table 1).

**Table 1:** Baseline characteristics of the study population

	All patients, *n* = 40	Control group, *n* = 20	Treatment group, *n* = 20	*P* value
Age, years	69 (51–76)	67 (53–75)	73 (49–77)	0.892
Female sex, *n* (%)	15 (38)	7 (35)	8 (40)	1.0
BMI	26 (24–28)	27 (24–29)	24 (23–26)	0.100
SAPS2	51 ± 16	47 ± 16	56 ± 14	0.055
PaO_2_/FiO_2_^a^	228 ± 94	214 ± 90	241 ± 99	0.375
PEEP^a^, cmH_2_O	10 (8–10)	10 (8–12)	9 (8–10)	0.104
Reason for admission, *n* (%)				0.112
Medical	28 (70)	11 (55)	17 (85)	
Elective surgery	5 (12)	4 (20)	1 (5)	
Emergency surgery	7 (18)	5 (25)	2 (10)	
ETT size, mm ID	7.5 (7.5–7.5)	7.5 (7.5–8.0)	7.5 (7.5–7.5)	0.113
Suspect of pneumonia, *n* (%)	13 (32)	6 (30)	7 (35)	1.0
ARDS diagnosis, *n* (%)	6 (15)	5 (25)	1 (5)	0.182

*BMI* body mass index, *SAPS2* Simplified acute physiology score II, *PEEP* positive end-expiratory pressure, *ETT* endotracheal tube, *ID* internal diameter, *ARDS* acute respiratory distress syndrome

^a^Measured at intubation

### Secretions within the ETT

**Fig. 2:**
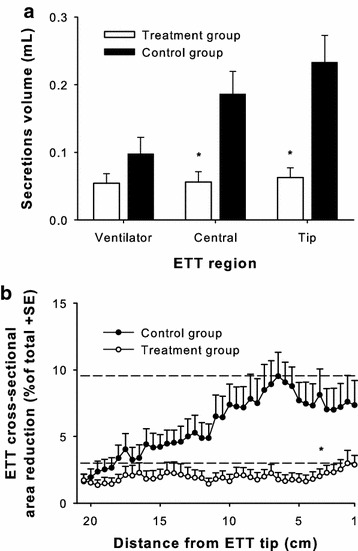
Efficacy of the ETT cleaning system. **a** The volume of ETT secretions increases in the control group (*black columns*) from the ventilator to the tip side (secretions analyzed dividing the studied ETT length in three equal parts), while it is lower (*p* < 0.001 by ANOVA) and fairly constant in the treatment group (*white columns*). **b** ETT cross-sectional area reduction measured every 5 mm among treatments (*white circles*) and controls (*black circles*). The maximum cross-sectional area reduction is markedly lower in the treatment group (*dashed lines*, *p* = 0.003). *p < 0.05 as compared to controls

The volume of secretions present in the ETT at extubation was reduced in the treatment group, as compared to controls (*p* = 0.001, Table 2). Correspondingly, the ETT cross-sectional area occupied by secretions was six times lower in the treatment group, as compared to controls (*p* = 0.001, Table 2). Secretions amount in the control group increased from the ventilator to the tip side, while in the treatment group the regional distribution of secretions along the ETT was different, resulting lower and fairly constant along the entire ETT (*p* < 0.001 for treatment effect, *p* = 0.005 for the interaction between the treatment effect and the region effect by ANOVA, see Fig. 2a for post hoc tests).

**Table 2:** ETT analysis after extubation

	All patients, *n* = 40	Control group, *n* = 20	Treatment group, *n* = 20	*P* value
Study ETT in place, days	5 (3–8)	5 (3–7)	5 (3–10)	0.512
ETT secretions, volume (mL)	0.274 (0.046–0.576)	0.568 (0.162–0.756)	0.081 (0.021–0.306)	0.001
Average CSA reduction (%)	3 (1–6)	6 (2–10)	1 (0–3)	0.001
Tracheal aspirate cultures, log (CFU)	0 (0–5)	0 (0–3)	0 (0–5)	0.234
ETT colonization, log (CFU)	2 (0–4)	2 (0–4)	1 (0–4)	0.568
Leukocytes in ETT lavage, n. pos. (%)	6 (15)	6 (30)	0 (0)	0.019

*ETT* endotracheal tube, *CSA* cross-sectional area, *CFU* colony forming units, *n. pos.* number of ETT lavage samples positive for leukocytes presence

Analyzing the local cross-sectional area reduction in ETT slices of 0.5-mm thickness, the peak average lumen reduction was lower in the treatment than in the control group (3 ± 4.4 vs. 9.5 ± 8 %, *p* = 0.003, Fig. 2b). Considering the maximum local cross-sectional area reduction, none of the ETTs in the treatment group showed a lumen reduction over 20 % (maximum cross-sectional area reduction 17 %) at the moment of ETT removal. Instead, half of the patients in the control group had their ETTs showing a maximum cross-sectional area reduction of more than 20 % of the nominal value (maximum 34 %, *p* < 0.001 for a cut-off of 20 % maximum cross-sectional area reduction in comparison with treatment group).

### Microbiological data

The total microbial growth of cultures obtained from the ETT lavages after extubation did not differ between the treatment and the control group, suggesting similar ETT colonization. Similarly, total microbial growth did not differ between the two groups considering the tracheal aspirate samples collected before extubation. However, leukocytes were present only in the ETT lavages of the control group (Table 2).

The VAP rate did not differ between the two groups (one VAP diagnosed in each group), as well as VAE retrospectively assessed. We recorded 4 vs. 1 VAC, 3 vs. 1 IVAC, 0 vs. 1 pVAP in the treatment and control group, respectively (all *p* > 0.05).

### Clinical data during ICU stay

Mortality at ICU discharge did not differ between the treatment and the control group, as well as ventilator free days evaluated after 28 days from admission and length of ICU stay among survivors (Additional file 1 Table 1, reporting patients’ outcomes on study completion). Several clinical variables related to lungs function and infection (such as PaO_2_/FiO_2_ ratio, mCPIS, see Table 3) did not differ between the two groups.

**Table 3:** Patient clinical course during ICU stay

	Day 1 treatment	Controls	Day 2 treatment	Controls	Day 3 treatment	Controls
mCPIS	3 (2–5)	3 (3–5)	3 (1–4)	3 (2–5)	3 (2–4)	3 (2–4)
PaO_2_/FiO_2_ ratio	240 ± 75	215 ± 80	250 ± 72	204 ± 74	226 ± 61	199 ± 76
Body temperature (°C)	37.0 (36.5–37.3)	37.1 (36.5–37.5)	37 (36.3–37.7)	37.0 (36.3–37.4)	37 (36.8–37.5)	37.1 (36.7–37.7)
WBC, *n* × 10^9^/L	8.8 (6.4–14.5)	9.2 (7.5–14.6)	10 (6.2–16.2)	10.4 (7.6–14.6)	8.3 (3.3–12.6)	8.5 (7.4–12)
PCT, ng/ml	7.4 (2.1–20.8)	1.8 (0.6–27.6)	2.8 (0.5–11.1)	1.8 (0.4–13.1)	1.91 (0.39–12.91)	1.2 (0.3–7.3)
RX score,* n* fields	1 (1–2)	2 (1–3)	2 (1–2)	2 (1–3)	2 (1–3)	2 (1–3)
TV, mL	500 ± 110	470 ± 100	430 ± 60	400 ± 90	460 ± 80	475 ± 140
PEEP, cmH_2_O	8 (7–10)	10 (7–12)	8 (6–10)	9 (8–11)	8 (7–10)	9 (8–13)
CPL, mL/cmH_2_O	46 (34–60)	46 (35–61)	47 (30–66)	41 (33-50)	50 (35–64)	44 (30–51)
Res, cmH_2_O·L^−1^·s^−1^	9 (9–11)	13 (10–15)	9 (8–11)*	15 (13–15)	9 (8–9)	9 (9)
CRP	11.6 (7.1–29.9)	17.1 (7.5–32.4)	16.6 (6.6–25.7)	15.4 (8.8–22.6)	15.5 (8.9–31.3)	9.6 (6.7–22.5)

*mCPIS* modified clinical pulmonary infection score, *WBC* white blood cells, *PCT* procalcitonin, *RX score* number of involved quadrants (0–4), *TV* tidal volume, *PEEP* positive end-expiratory pressure, *CPL* compliance, *Res* resistance to airflow, *CRP* c-reactive protein

* *p* < 0.05 as compared to control group

During ICU stay, the need of suctioning (median daily suction maneuvers after enrollment) tended to be lower in the treatment group as compared to controls (3 [2–4] vs. 5 [3–7], *p* = 0.068), while we recorded no difference between the two groups at enrollment (3 [1–4] vs. 3 [2–6], *p* = 0.463). The number of patients with one or more recordings of macroscopic presence of blood in the tracheal aspirates tended to be lower in the treatment than in the control group (2/20 vs. 7/20, *p* = 0.058 by Chi-square test). In the subset of patients (8 treatments and 8 controls) ventilated in controlled modes for at least 24 h, we measured airways resistance daily, until switch to assisted modes of ventilation. Considering the first 3 days of ICU stay after enrollment, resistance to airflow during intubation tended to be lower in the treatment group as compared to controls on day 1 (*p* = 0.063, Table 3), was lower on day 2 (0.024), and did not differ on day 3 (*p* = 0.317), while no difference was present at enrollment (*p* = 0.922). As in the general study population, the ETT size did not differ between the two groups in this subset of patients (*p* = 0.798).

## Discussion

In this randomized clinical trial, we showed that the regular use of a cleaning CSS is effective to reduce the volume of secretions present in the ETT at extubation, as assessed by MicroCT scan. We evaluated the effectiveness of a novel device in the everyday practice of a general ICU, excluding patients whose risk of ETT displacement was too high for safety reasons, since we had no previous data available about the clinical use of the cleaning device. The presented data suggest that no specific limitation should be present for the use of such a cleaning CSS. We chose the volume of secretion present at extubation as the primary endpoint because the device was primarily designed to remove secretions, either by aspiration or cleaning. We reasoned that establishing its efficacy in this respect should be preliminary to investigating any clinical endpoint. Moreover, the volume of secretions in the ETT was an endpoint which we could assess with high precision, as previously described by our group using a novel technique such as MicroCT scan [8]. MicroCT, in comparison with other techniques proposed for the measurement of the amount of secretions within the ETT, allowed us to directly study a large portion of the ETT (20 cm) with a fairly high resolution (35 μm).

The device we used is not the first cleaning system based on the retraction of an inflated balloon. Other similar devices were studied in the past, and showed good effectiveness [13, 14]. However, those devices were designed with the sole purpose of cleaning the ETT, requiring patient disconnection from the ventilator circuit. The novelty of the device used in this study is that the cleaning system is embedded in a closed suction system resembling the one commonly used in our ICU for tracheal suction in patients expected to be ventilated for several days. The advantage of using a closed system is that patient disconnection is not required to perform both suction and cleaning maneuvers, and positive pressure is preserved in the airways. Moreover, airways contamination is reduced, and some guidelines recommend the use of such systems [15–18]. Thus, the study device offered features similar to the standard of care in our ICU, with the added benefit of ETT cleaning.

The patients enrolled in the study were representative of a general ICU population, including both surgical and medical patients. The analyzed ETTs were similar in the two groups, both in terms of size (Table 1) and number of days they remained in place (median 5 days, Table 2). In the control group, we report a cross-sectional area reduction similar to data available in the literature [8, 19, 20]. Despite the use of the cleaning device, the ETTs in the treatment group did not result completely free from secretions, for several reasons. First, the efficacy of the cleaning system might not be optimal in some patients, possibly due to different physical properties of the secretions (i.e., density, adhesion to the plastic surface) and to the presence of an amount of biofilm which is difficult to remove [21]. Second, in some patients, more than three cleaning maneuvers might be needed to preserve patency of the ETT. Future studies might investigate if a different protocol based on patients’ needs (i.e., equal number of cleaning and suction maneuvers) is more effective. Third, we did not clean the ETT immediately before extubation, and some secretions might have gathered within the ETT after the last cleaning maneuver was performed or during the extubation procedure. We decided not to perform a cleaning maneuver before extubation to have a better understanding of the effects of the cleaning device on ETT patency during a real ICU shift, rather than immediately after cleaning, adding strength to our results.

As we previously showed, the tip side is the part where secretions tend to collect, both because of the proximity with trachea, where secretions gather, and because that ETT part is the more dependent when the patient lies in the semi-recumbent position. We showed that the lack of secretion in the proximal, visible part of the ETT does not exclude a relevant loss of cross-sectional area in the endotracheal portion, whose presence can be clinically disclosed only by bronchoscopy. We showed that the use of a cleaning device reduces the loss of internal diameter, and the preventive effect is particularly evident in the tip side. This finding is consistent with the finding of reduced airflow resistance associated with the use of the novel CSS.

We could not demonstrate any difference in ETT pathogen colonization between the treatment and the control groups, a finding previously reported with other ETT cleaning devices [7]. The cleaning balloon is designed to maintain ETT patency, but a thin layer of contaminated biofilm could remain on the ETT surface resulting in positive cultures when colonization is assessed by lavage of the entire ETT. Future studies might investigate if ETT colonization can be reduced by cleaning balloons of different shape or using moisturizing solution other than saline. However, we report a reduced amount of leukocytes in ETT lavage fluids obtained from the treatment group. We might speculate that the use of a cleaning device results in a reduced inflammatory stimulation of the mucosa, reflected by a lower amount of leukocytes in the ETT biofilm, lower mucosal fragility and reduced production of secretions, leading to a lower need for suction and less mucosal injury. Such findings, if confirmed, might be clinically relevant because lower production of secretions can lead to lower risk of sudden ETT obstruction and lower increase of airways resistance due to loss of ETT patency.

Regarding the major clinical outcomes, the present study was neither aimed, nor powered to demonstrate differences in mortality, ICU ventilator free days or VAP/VAE incidence. Similarly, we could not find any difference in mCPIS, a clinical score associated with presence of lung infection. A larger population is needed to investigate those outcomes, and is debatable if a single component of ETT care, such as a cleaning catheter, is able to significantly impact the clinical course of critically ill patients. Based on the results of this study, the use of a cleaning catheter appears safe and no relevant contraindication to its use emerged. However, the relevance of the endpoints investigated in this study is not sufficient to mandate a change in the current practice and a routine use of such cleaning catheters in every intubated patient.

## Conclusions

In this randomized clinical study, we report that the regular use of a cleaning CSS is effective in reducing the amount of secretions present in the ETT at extubation, particularly in the part closer to the trachea. The reduction of secretions in the ETT was associated with lower resistance to airflow in a subset of patients. We report no relevant adverse events related to the use of the cleaning device other than the ones known to be associated with standard suction. Studies on larger populations are needed to prove the relevance of the use of a cleaning catheter on major clinical outcomes.

## Additional file

10.1186/s13613-015-0101-9 Supplemental Methods and Outcome table.

## Abbreviations

ETT: endotracheal tube
VAP: ventilator-associated pneumonia
CSS: closed suction system
MicroCT: micro-computed tomography
ICU: intensive care unit
SDC: Supplemental digital content
mCPIS: modified clinical pulmonary infection score
VAE: ventilator-associated events

## References

[1] Blanc V, Tremblay N (1974). The complications of tracheal intubation: a new classification with a review of the literature. Anesth Analg.

[2] Sottile F, Marrie T, Prough D, Hobgood C, Gower D, Webb L (1986). Nosocomial pulmonary infection: possible etiologic significance of bacterial adhesion to endotracheal tubes. Crit Care Med.

[3] Inglis TJ, Lim TM, Ng ML, Tang EK, Hui KP (1995). Structural features of tracheal tube biofilm formed during prolonged mechanical ventilation. Chest.

[4] Inglis T, Millar M, Jones J, Robinson D (1989). Tracheal tube biofilm as a source of bacterial colonization of the lung. J Clin Microbiol.

[5] Kollef MH, Afessa B, Anzueto A, Veremakis C, Kerr KM, Margolis BD (2008). Silver-coated endotracheal tubes and incidence of ventilator-associated pneumonia: the NASCENT randomized trial. JAMA.

[6] Coppadoro A, Bittner E, Berra L (2012). Novel preventive strategies for ventilator-associated pneumonia. Crit Care.

[7] Berra L, Coppadoro A, Bittner E, Kolobow T, Laquerriere P, Pohlmann J (2012). A clinical assessment of the mucus shaver: a device to keep the endotracheal tube free from secretions. Crit Care Med.

[8] Coppadoro A, Bellani G, Bronco A, Borsa R, Lucchini A, Bramati S (2014). Measurement of endotracheal tube secretions volume by micro computed tomography (MicroCT) scan: an experimental and clinical study. BMC anesthesiol.

[9] Lucchini A, Zanella A, Bellani G, Gariboldi R, Foti G, Pesenti A (2011). Tracheal secretion management in the mechanically ventilated patient: comparison of standard assessment and an acoustic secretion detector. Respir Care.

[10] Ranieri VM, Rubenfeld GD, Thompson BT, Ferguson ND, Caldwell E, Fan E (2012). Acute respiratory distress syndrome: the Berlin definition. JAMA.

[11] Le Gall JR, Lemeshow S, Saulnier F (1993). A new simplified acute physiology score (SAPS II) based on a European/North American multicenter study. JAMA.

[12] Fartoukh M, Maitre B, Honore S, Cerf C, Zahar JR, Brun-Buisson C (2002). Diagnosing pneumonia during mechanical ventilation: the clinical pulmonary infection score revisited. Am J Respir Crit Care Med.

[13] Conti G, Rocco M, De Blasi RA, Lappa A, Antonelli M, Bufi M (1994). A new device to remove obstruction from endotracheal tubes during mechanical ventilation in critically ill patients. Intensive Care Med.

[14] Kolobow T, Berra L, Li Bassi G, Curto F (2005). Novel system for complete removal of secretions within the endotracheal tube: the mucus shaver. Anesthesiology.

[15] Rabitsch W, Kostler WJ, Fiebiger W, Dielacher C, Losert H, Sherif C (2004). Closed suctioning system reduces cross-contamination between bronchial system and gastric juices. Anesth Analg.

[16] Siempos II, Vardakas KZ, Falagas ME (2008). Closed tracheal suction systems for prevention of ventilator-associated pneumonia. Br J Anaesth.

[17] Hess DR, Kallstrom TJ, Mottram CD, Myers TR, Sorenson HM, Vines DL (2003). Care of the ventilator circuit and its relation to ventilator-associated pneumonia. Respir Care.

[18] Maggiore SM, Lellouche F, Pignataro C, Girou E, Maitre B, Richard JC (2013). Decreasing the adverse effects of endotracheal suctioning during mechanical ventilation by changing practice. Respir Care.

[19] Mietto C, Pinciroli R, Piriyapatsom A, Thomas JG, Bry L, Delaney ML (2014). Tracheal tube obstruction in mechanically ventilated patients assessed by high-resolution computed tomography. Anesthesiology.

[20] Shah C, Kollef MH (2004). Endotracheal tube intraluminal volume loss among mechanically ventilated patients. Crit Care Med.

[21] Danin PE, Girou E, Legrand P, Louis B, Fodil R, Christov C (2015). Description and microbiology of endotracheal tube biofilm in mechanically ventilated subjects. Respir Care.

