# Supplementary material for: The use of a novel cleaning closed suction system reduces the volume of secretions within the endotracheal tube as assessed by micro-computed tomography: a randomized clinical trial
Source: Ann Intensive Care. 2015 Dec 30;5:57. doi: 10.1186/s13613-015-0101-9 (PMC4695481; doi:10.1186/s13613-015-0101-9)
Supplement: Supplementary file 1 — 10.1186/s13613-015-0101-9 Supplemental Methods and Outcome table. [file 13613_2015_101_MOESM1_ESM.pdf]

Supplemental digital content (SDC) for the study entitled: The use of a novel cleaning closed suction system reduces the volume of secretions within the endotracheal tube as assessed by micro-computed tomography: a randomized clinical trial

Coppadoro A[1]*, Bellani G[1,2], Bronco A[1], Lucchini A[2], Bramati S[3], Zambelli V[1], Marcolin R[2], Pesenti A[1,2]

[1] Department of Health Science, University of Milan-Bicocca, Monza, Italy

[2] Department of Anesthesia and Intensive care, San Gerardo Hospital, Monza, Italy

[3] Laboratory of Microbiology San Gerardo Hospital, Monza, Italy

* Current affiliation: A.Manzoni Hospital, Lecco, Italy

Methods

Study protocol

The novel cleaning CSS, as well as the standard CSS used in the control group, was managed by the critical care nurse in charge of the patient. Nurses were trained on the use of the novel CSS by explanation of the use and a mannequin bench demonstration. Reasons to initiate a tracheal suction maneuver included: coarse breath sounds on auscultation, dyspnea, cough, apparent increased work of breathing, increased inspiratory peak pressure during volume-controlled ventilation, decreased tidal volume during pressure-controlled ventilation, SpO2 decrease not otherwise explained, abnormalities on the expiratory flow trace on the ventilator, visible secretions in the endotracheal tube or ventilator circuit, or alert from the secretions-detector (TBA Care system, First Medica, Cusago, Italy). Pre-oxygenation before suction was routinely used. Heated humidifiers were used for all patients. Any adverse effect possibly related with the use of the cleaning CSS was recorded. In case of extubation and subsequent re-intubation, the enrolled patients were studied only once.

Data collection

At enrolment, we collected some clinical parameters to characterize the study population (PaO_2/FiO_2 ratio, PEEP set at the ventilator, ETT size, presence of Acute Respiratory Distress Syndrome diagnosis (10), Simplified Acute Physiology Score II [SAPS2])(11). During ICU stay, every day we collected clinical data regarding ventilation, (PaO2, FiO2, PEEP, Tidal volume), respiratory mechanics measurements such as compliance of the respiratory system (tidal volume/driving

pressure), resistance to airflow (peak – plateau pressure /ventilatory flow) and possible signs of infection (visual assessment of aspirated secretions, white blood cells concentration, body temperature, chest x-ray number of quadrants involved [RX score, 1 to 4 quadrants]) to calculate a modified clinical pulmonary infection score (mCPIS)(12). C-reactive protein and procalcitonin plasma concentration were also recorded when the exam was requested for clinical purpose. When feasible, we collected surveillance tracheal aspirate samples in the 24 hours prior to extubation. At extubation, we recorded ICU length of stay, ventilator free days in the first 28 days and patient outcome. Ventilator associated pneumonia (VAP) was defined as the presence of new infiltrates on chest x-ray after >48h from intubation, together with at least two of the following: body temperature >38°C, white blood cells count >12,000 or <4,000 cells/mm3 and/or purulent tracheobronchial secretions; as well as with a positive BAL culture. Retrospectively, we also assessed the number of ventilator associated events (VAE), distinguishing in ventilator associated condition (VAC), infection-related ventilator-associated complication (IVAC) and possible/probable VAP (pVAP).

Sample processing

Right after extubation, the ETTs were sealed on both sides to prevent external microbial contamination and drying of secretions. To assess ETT microbial colonization, we injected in a sterile fashion 5 ml of saline solution to collect lavage fluids of the ETT inner lumen. The obtained samples were sent to the microbiology lab of our hospital and microbiological growth was recorded. The number of leukocytes in the samples was assessed by the microbiology lab following the standard procedures used for clinical samples. To analyze differences between samples collected in the treatment and the control group, the total microbial growth was considered (i.e. sum of Log [colony forming units] for each microbe recorded).

Table 1 ESM. Patient outcome on study completion

	All patients n=40	Control group n=20	Treatment Group n=20	p-value
28-day ventilator free days, n.	20 [0-25]	21 [2-25]	8 [0-24]	0.165
ICU Mortality, n. (%)	18 (45)	6 (30)	12 (60)	0.111
Survivors ICU stay (days)	10 [6-18]	13 [6-18]	7 [4-19]	0.267

ICU, intensive care unit

10. Ranieri VM, Rubenfeld GD, Thompson BT, Ferguson ND, Caldwell E, Fan E et al. Acute respiratory distress syndrome: the Berlin Definition. Jama. 2012;307:2526-33. doi:10.1001/jama.2012.5669
1160659 [pii].
11. Le Gall JR, Lemeshow S, Saulnier F. A new Simplified Acute Physiology Score (SAPS II) based on a European/North American multicenter study. Jama. 1993;270:2957-63.
12. Fartoukh M, Maitre B, Honore S, Cerf C, Zahar JR, Brun-Buisson C. Diagnosing pneumonia during mechanical ventilation: the clinical pulmonary infection score revisited. Am J Respir Crit Care Med. 2003;168:173-9. doi:10.1164/rccm.200212-1449OC
200212-1449OC [pii].
